# Dual PD-1 and CTLA-4 targeting in endometrial carcinoma: integrating efficacy, toxicity, and biomarkers into clinical practice

**DOI:** 10.3389/fimmu.2026.1771548

**Published:** 2026-02-27

**Authors:** Lan Zhong, Liang Song

**Affiliations:** 1 Department of Obstetrics and Gynecology, West China Second University Hospital, Sichuan University, Chengdu, Sichuan, China; 2 Key Laboratory of Birth Defects and Related Diseases of Women and Children (Sichuan University), Ministry of Education, Chengdu, China

**Keywords:** biomarkers, endometrial carcinoma, immune checkpoint inhibitors, immunotherapy, treatment-related adverse events

## Abstract

The management of advanced endometrial cancer (EC) has been transformed by immunotherapy, raising a pivotal clinical challenge: selecting patients with mismatch repair–deficient (dMMR) disease for intensive dual PD-1/CTLA-4 blockade versus standard PD-1 monotherapy. We conducted a narrative review of phase II/III clinical trials and key translational studies published up to 2023 to critically appraise current evidence. In dMMR EC, the conventional ipilimumab-nivolumab combination yields higher objective response rates (ORR ≈ 63%) than PD-1 monotherapy (ORR ≈ 48%) but is associated with a substantially increased incidence of grade ≥ 3 immune-related adverse events (≈ 23% vs. ≈ 12%). The development of bispecific antibodies like cadonilimab, which demonstrates robust efficacy with a lower incidence of high-grade toxicity (grade ≥ 3 treatment-related adverse events: 8.3%), presents a promising strategy to improve the therapeutic index. For clinicians, the current decision-making process must be highly individualized, weighing factors such as tumor burden, pace of disease, and patient tolerance for toxicity in the absence of validated biomarkers to guide treatment intensity beyond dMMR status. We also addressed the critical importance of accurate MMR/MSI testing and the clinical implications of a well-documented methodological discordance rate. In contrast, for patients with mismatch repair–proficient (pMMR) tumors, the evidence firmly supports alternative regimens, such as lenvatinib plus pembrolizumab, over dual PD-1/CTLA-4 blockade. Navigating the evolving landscape of immunotherapy in EC requires a nuanced, patient-centric approach. The integration of novel bispecific antibodies may soon simplify the balance between efficacy and toxicity, but until then, treatment selection remains a deliberate process, underscoring the gynecologic oncologist’s pivotal role in personalizing care.

## Highlights

- dMMR EC: Dual PD-1/CTLA-4 blockade improves response rates but increases toxicity versus PD-1 monotherapy.
- Bispecific antibodies (e.g., cadonilimab) show improved efficacy-toxicity profiles, simplifying treatment choice.
- Individualized decisions integrating tumor burden & progression kinetics are needed without predictive biomarkers.
- MMR/MSI testing discordance necessitates confirmatory strategies for accurate patient identification.
- In pMMR EC, dual checkpoint blockade has limited efficacy;lenvatinib/pembrolizumab remains standard.

## 1 Introduction

Endometrial carcinoma remains the most prevalent gynecologic malignancy in developed regions. Its increasing incidence rates underscore the urgent need for advanced therapeutic strategies tailored to improve patient outcomes (1). The molecular classification system established by The Cancer Genome Atlas has evolved from a prognostic framework to a clear guide for treatment selection (2). The mismatch repair–deficient (dMMR) or microsatellite instability–high (MSI-H) molecular subgroup comprises approximately 30% of endometrial cancer cases. This subgroup exhibits a high tumor mutational burden and an immunologically active microenvironment, making it particularly susceptible to immune checkpoint inhibition (3).

Regulatory approval of anti–PD-1 antibodies, including pembrolizumab and dostarlimab, has established a new therapeutic paradigm for advanced mismatch repair-deficient/microsatellite instability-high (dMMR/MSI-H) endometrial cancer (EC), demonstrating durable responses and manageable toxicity profiles (4, 5). This success prompted investigations of more intensive immunotherapy regimens. The combination of PD-1 and CTLA-4 blockade has emerged as a highly effective strategy for advanced endometrial cancer, yet it introduces a fundamental and unresolved clinical dilemma: determining which patients will derive sufficient benefit from this approach to justify the substantial increase in toxicity risks. Although PD-L1 inhibitors such as durvalumab and atezolizumab have shown activity in other solid tumors and are under investigation in EC, the most robust clinical evidence currently supports PD-1/CTLA-4 targeting strategies in EC. This preference is based on the non-redundant biological roles of the PD-1 and CTLA-4 pathways and the demonstrated clinical synergy of their combined inhibition in dMMR/MSI-H tumors across multiple cancer types. Direct comparisons between PD-1/CTLA-4 combinations and PD-L1 inhibitors in EC are lacking, but the PD-1/CTLA-4 combination approach has generated more extensive efficacy data specifically in endometrial carcinoma. This review critically evaluates the current evidence for traditional combinations and emerging bispecific antibodies, to address this pivotal question, while underscoring the essential role of the gynecologic oncologist in navigating these complex therapeutic decisions in the absence of definitive predictive biomarkers.

## 2 Biological rationale for combination therapy

The therapeutic synergy between inhibition of PD-1 and CTLA-4 stems from their complementary roles in T-cell regulation. CTLA-4 primarily functions in lymphoid organs during early T-cell activation, modulating the magnitude of initial immune responses. Inhibition of CTLA-4 promotes T-cell repertoire diversification and expansion, thereby potentially recruiting novel anti-tumor clones (6). Conversely, the PD-1 pathway operates predominantly in peripheral tissues where it attenuates effector functions of tumor-infiltrating lymphocytes, resulting in a state of functional exhaustion. PD-1 blockade serves to reinvigorate these pre-existing antitumor T-cell populations within the tumor microenvironment (7).

This immunobiological framework aligns with the observed heterogeneity of the endometrial cancer tumor microenvironment. dMMR/MSI-H tumors typically display an immune-inflamed phenotype, characterized by abundant cytotoxic T-cell infiltration and PD-L1 expression, thereby providing the necessary immune context for effective PD-1 blockade therapy (3, 8). In contrast, many mismatch repair–proficient/microsatellite stable (pMMR/MSS) tumors exhibit immune-excluded or immune-desert phenotypes, suggesting deficiencies in initial T-cell priming and trafficking. These processes can potentially be modulated through CTLA-4 inhibition (9). This spectrum of ‘immune-hot’ to ‘immune-cold’ phenotypes, as conceptualized in the cancer-immunity cycle (10), provides a strategic framework for rational combination immunotherapy. Thus, the combination strategy concurrently targets both the initiation and effector phases of the cancer-immunity cycle, a principle supported by the efficacy of the nivolumab and ipilimumab combination therapy in tumors with a high mutational burden across cancer types (11).

## 3 Clinical evidence and therapeutic applications

### 3.1 dMMR/MSI-H population: weighing intensity against toxicity

The clinical efficacy of the ipilimumab-nivolumab combination in dMMR/MSI-H endometrial cancer is primarily supported by a recent basket trial reported by Carlino et al. (12), which represents the only dedicated phase II study evaluating this specific combination in dMMR/MSI-H EC. In the prespecified endometrial cancer subgroup of this trial (n=26), the regimen demonstrated an objective response rate (ORR) of 58% (95% CI, 39–74) and a disease control rate (DCR) of 77% (95% CI, 58–89). While other basket trials (e.g., CheckMate 142) have included small EC cohorts, they were not powered for subtype-specific analysis (13). The observed efficacy is consistent with the established, potent activity of dual PD-1/CTLA-4 blockade in other dMMR/MSI-H solid tumors, such as metastatic colorectal cancer (13).

This ORR is numerically higher than the 48.5% (95% CI, 36.2–61.0) reported for pembrolizumab monotherapy in the KEYNOTE-158 endometrial cancer cohort (4). However, such cross-trial comparisons must be interpreted with caution. Notably, the current standard first-line therapy for advanced EC, regardless of MMR status, is increasingly shifting toward chemo-immunotherapy combinations. The phase III NRG-GY018 trial established that adding pembrolizumab to carboplatin and paclitaxel significantly improved progression-free survival in both dMMR and pMMR cohorts (21). In the dMMR subgroup, the hazard ratio for progression-free survival was 0.30 (95% CI, 0.19–0.48), demonstrating exceptional efficacy of this combination. However, there are currently no direct head-to-head trials comparing the ipilimumab-nivolumab regimen with chemo-immunotherapy in dMMR EC. Therefore, the current decision to employ this intensive regimen must carefully weigh a substantial and well-documented increase in toxicity against an incremental benefit over standard PD-1 monotherapy whose magnitude remains unquantified, while acknowledging the emerging role of first-line chemo-immunotherapy. It is also important to note that, while molecular classification guides therapy, prospective data on dual checkpoint blockade specifically stratified by TCGA molecular subgroups are limited in availability, with current evidence primarily based on MMR status. Definitive evidence establishing the superiority of the combination over PD-1 inhibitor monotherapy in endometrial cancer is awaited from the ongoing phase III NRG-GY025 trial.

### 3.2 Bispecific antibodies: an engineered approach to dual blockade

The logistical and toxicity challenges associated with co-administering two separate monoclonal antibodies have spurred the development of innovative, engineered molecules designed for coordinated dual targeting, aiming to improve the therapeutic index.

Cadonilimab (AK104) is a tetravalent bispecific antibody that targets both PD-1 and CTLA-4 with a single molecular entity. Its design, featuring a higher affinity for PD-1, aims to achieve preferential dual immune checkpoint blockade within the tumor microenvironment (TME). While CTLA-4 primarily functions in secondary lymphoid organs during early T-cell priming, the bispecific design of cadonilimab is theorized to concentrate dual blockade of PD-1 and CTLA-4 within the tumor microenvironment (TME). This approach potentially reduces systemic CTLA-4–mediated toxicities while still allowing for CTLA-4 inhibition on tumor-infiltrating lymphocytes and antigen-presenting cells that have migrated to the tumor site (14). This localized activity may mitigate the systemic CTLA-4–related toxicities often seen with conventional combination therapy by sparing excessive peripheral T-cell activation (14). Early-phase clinical trials of cadonilimab have demonstrated antitumor activity and a manageable safety profile across a spectrum of advanced solid tumors (15). A recent systematic review and meta-analysis reported a pooled incidence of grade ≥3 treatment-related adverse events of 11.3% (95% CI, 9.5–13.3) (16). The consistency of this favorable safety data across studies, characterized by a lower rate of high-grade toxicities than typically associated with conventional CTLA-4–containing combinations, supports the premise that cadonilimab may offer an improved therapeutic index.

Recent phase II evidence has further solidified the potential of cadonilimab in endometrial carcinoma. A multicenter, single-arm trial evaluated cadonilimab in combination with lenvatinib as a second- or later-line therapy for patients with advanced endometrial cancer who progressed after platinum-based chemotherapy (17). Among 32 enrolled patients, the objective response rate was 37.5% (12/32) in the full analysis set and 42.9% (12/28) in the efficacy-evaluable set, with a compelling disease control rate of 81.3% and 92.9%, respectively. Notably, the median progression-free survival was not reached after a median follow-up of 7.6 months. The combination exhibited a manageable safety profile, with grade 3 or higher treatment-related adverse events occurring in 21.9% of patients; the most common were intestinal obstruction (6.3%), increased alanine aminotransferase (6.3%), and hypertension (3.1%). This study provides preliminary but encouraging clinical evidence for the cadonilimab-lenvatinib combination in a dedicated endometrial cancer cohort. However, these findings should be interpreted considering the current evidence base, which is derived primarily from single-arm studies. The absence of randomized comparative data necessitates cautious interpretation.

A distinct technological approach is embodied by QL1706, a fixed-ratio combination of two monoclonal antibodies produced using the MabPair^®^ platform. This technology enables the co-expression of both antibodies from a single cell line, ensuring a consistent product ratio and quality (18). A key pharmacokinetic feature is the engineered shorter elimination half-life of the anti–CTLA-4 component, designed to reduce its systemic exposure and potentially lower the risk of immune-related adverse events. This unique profile, which maintains sustained PD-1 blockade while limiting CTLA-4 exposure, aims to improve tolerability and facilitate longer treatment durations (18).

Together, bispecific and combination antibodies represent a shift toward the rational design of integrated dual-targeting agents. The goal of this approach is to enhance antitumor efficacy by concurrently engaging complementary immune pathways while aiming to improve the safety profile through optimized pharmacokinetics and tumor-targeted activity.

### 3.3 pMMR/MSS population: current therapeutic limitations and future directions

The efficacy of PD-1 inhibitor monotherapy is markedly limited in advanced pMMR/MSS endometrial cancer, with objective response rates consistently below 15%. In the KEYNOTE-158 endometrial cancer cohort, the ORR was 6.5% (95% CI, 2.2–14.5) in pMMR patients (4), while in the GARNET study, dostarlimab monotherapy achieved an ORR of 14.1% in the pMMR/MSS population (5). This finding reflects the immunologically quiescent tumor microenvironment characteristic of this molecular subgroup (19). Similar challenges in overcoming the immunosuppressive milieu of pMMR tumors are noted across other cancer types, highlighting a common therapeutic hurdle (23). This fundamental limitation has redirected therapeutic strategies toward rational combinations capable of inducing inflammatory changes within the tumor immune landscape (20).

The clinical success of this paradigm is robustly supported by two landmark regimens. In the first-line setting, the phase III NRG-GY018 trial established that adding pembrolizumab to carboplatin and paclitaxel significantly improved progression-free survival (PFS) in the pMMR cohort. The hazard ratio (HR) for PFS was 0.64 (95% CI, 0.49–0.85), confirming a substantial reduction in the risk of disease progression or death with the combination therapy (21). Similarly, in the later-line setting, the combination of lenvatinib with pembrolizumab demonstrated superior efficacy over chemotherapy (22).

The limited clinical efficacy observed to date with dual PD-1/CTLA-4 blockade in pMMR/MSS endometrial cancer underscores the profound immunosuppression characteristic of this molecular subgroup and indicates that this specific immunotherapeutic strategy may be insufficient to overcome the associated resistance mechanisms. Despite these results, the compelling biological rationale—founded on the non-redundant roles of CTLA-4 and PD-1 inhibition (11)—continues to motivate the investigation of this approach. Future studies in pMMR/MSS endometrial cancer should prioritize the integration of predictive biomarkers to identify patient subsets that may derive benefit from this intensive immunotherapy approach.

### 3.4 Emerging evidence in specific histologic subtypes

The therapeutic landscape of dual immune checkpoint blockade continues to evolve. Research is expanding to explore activity in rare histologic subtypes. Clear cell carcinoma of the endometrium, which is predominantly mismatch repair proficient/microsatellite stable (pMMR/MSS) and portends a poor prognosis (24), represents one such rare histologic subtype of interest. A recent nonrandomized clinical trial evaluated the activity of nivolumab plus ipilimumab in a cohort of patients with advanced clear cell carcinomas, which included both ovarian and endometrial primaries (25). In the overall population (N = 28), the objective response rate (ORR) was 54% (95% CI, 35–71). Treatment efficacy appeared consistent across primary sites, with an ORR of 50% (95% CI, 9–91) in the smaller endometrial clear cell carcinoma subgroup. These findings are supported by preliminary data from other studies: the BrUOG 354 trial (presented at ASCO 2024) evaluated nivolumab plus ipilimumab in recurrent gynecologic clear cell carcinomas and reported encouraging activity, while the biomarker-driven MoST-CIRCUIT trial (presented at ESMO 2024) demonstrated preliminary evidence of efficacy for dual checkpoint blockade in rare tumors, including clear cell histology (26, 27). However, the small number of patients with endometrial primaries in these studies precludes definitive conclusions. This underscores the necessity for future studies with larger, histology-specific cohorts.

## 4 Clinical implementation challenges

### 4.1 The unresolved quest for predictive biomarkers

The central challenge in dMMR/MSI-H endometrial cancer management remains the absence of validated biomarkers to stratify patients according to treatment intensity. Although dMMR status generally predicts immunotherapy response reliably, it cannot consistently identify patients who require combination therapy versus those who achieve optimal outcomes with monotherapy. As emphasized by Cosgrove, exploratory analyses have not identified consistent genomic correlates—such as JAK1/2 and B2M alterations—that differentiate these patient subsets (28).

Tumor mutational burden (TMB) and PD-L1 expression have been extensively investigated as potential predictive biomarkers. In KEYNOTE-158, a TMB cutoff of ≥10 mutations/megabase was associated with improved response to pembrolizumab across tumor types; however, this biomarker lacks standardization and validation specifically in endometrial cancer (29). Similarly, PD-L1 expression by combined positive score (CPS) shows variable association with response in endometrial cancer, with some studies suggesting predictive value, while others show responses in PD-L1-negative tumors (30). Furthermore, JAK1/2 mutations and B2M loss, which can lead to resistance to interferon signaling and antigen presentation, respectively, have been implicated in primary resistance to checkpoint blockade, but these alterations are not routinely assessed in clinical practice.

Therefore, the development and validation of robust predictive biomarkers—potentially integrating genomic, transcriptomic, and microenvironmental features—is not merely an academic pursuit but an urgent clinical necessity. Achieving true personalization of immunotherapy intensity depends on this advancement.

### 4.2 Navigating toxicity and aiding patient decision-making

#### 4.2.1 Spectrum and incidence of immune-related adverse events

The toxicity profile of conventional ipilimumab-nivolumab therapy represents its principal limitation. In the Carlino et al. trial (12), grade ≥3 immune-related adverse events (irAEs) occurred in 23% of patients receiving ipilimumab-nivolumab (**Table 1**, Part B). This incidence is substantially higher than the 12.0% observed with pembrolizumab monotherapy in KEYNOTE-158 (4) (**Table 1**, Part B). Moreover, the spectrum of these severe toxicities differs qualitatively. The combination regimen is associated with higher incidences of colitis (7–10%), hepatitis (5–7%), rash (3–5%), and endocrinopathies such as hypophysitis (2–4%) and thyroiditis (2–3%), typically emerging within 6–12 weeks (**Table 1**, Part A). In contrast, PD-1 monotherapy exhibits a lower incidence of these events—particularly colitis and hypophysitis—which are more strongly linked to CTLA-4 inhibition (**Table 1**, Part A).

**Table 1:** Safety profile of PD-1/CTLA-4-targeted therapies in endometrial carcinoma.

Part A: Incidence and management of common Grade ≥3 Immune-Related Adverse Events (irAEs)				
Adverse event	Ipilimumab-Nivolumab (Grade ≥3)	Pembrolizumab monotherapy (Grade ≥3)	Typical onset (weeks)	Key management principles
Colitis	7-10%	1-2%	6-8	Corticosteroids, infliximab for steroid-refractory cases.
Hepatitis	5-7%	1-3%	6-12	Corticosteroids, mycophenolate mofetil if severe.
Rash	3-5%	1-2%	2-4	Topical/systemic corticosteroids, antihistamines.
Hypophysitis	2-4%	<1%	8-12	Hormone replacement, corticosteroids for acute inflammation.
Thyroiditis	2-3%	1-2%	6-12	Thyroid hormone replacement, rarely corticosteroids.
Pneumonitis	1-2%	1-2%	Variable	Corticosteroids, infliximab/cyclophosphamide if severe.
Part B: Overall safety parameters				
Safety parameter		Ipilimumab-Nivolumab combination		Pembrolizumab monotherapy
Study Population (Trial)		52 patients (Carlino et al. *JAMA Oncol*.) (12)		351 patients (KEYNOTE-158, Cohort K) (4)
Incidence of Any-Grade irAEs		75% (39/52)		19.7% (69/351)*
Incidence of Grade ≥3 irAEs		23% (12/52)		12.0% (42/351)
Most Common Grade ≥3 irAEs		Hepatitis (6%), Enterocolitis (6%)		Pneumonitis (0.9%), Colitis (0.3%)
Treatment Discontinuation Due to irAEs		9.6% (5/52)		6.6% (23/351)
Fatal irAEs		Not reported		0.9% (3/351)

Data synthesized from Carlino et al. (2025) (12) and KEYNOTE-158 (O’Malley et al., 2022) (4).

1. Ipilimumab-Nivolumab data are derived from the phase 2 MOST-CIRCUIT trial in advanced dMMR/MSI-H noncolorectal cancers, where endometrial carcinoma represented 50% of the cohort (12).

2. Pembrolizumab data are from the phase 2 KEYNOTE-158 study (Cohort K) in previously treated advanced MSI-H/dMMR noncolorectal cancers, which included 22.5% endometrial carcinoma (4).

3. *The reported 19.7% refers specifically to “immune-mediated AEs” in KEYNOTE-158; treatment-related AEs of any grade occurred in 64.7% of patients (4).

4. Typical Onset represents the median or most frequent range from treatment initiation.

5. Thyroiditis in the monotherapy cohort primarily encompassed hypothyroidism (8.8% any grade) and hyperthyroidism (4.3% any grade), with minimal Grade ≥3 events (4).

Management principles are aligned with contemporary ESMO clinical practice guidelines.

In this context, a systematic review and meta-analysis specifically addressing the safety of cadonilimab reported a pooled incidence of grade ≥3 irAEs of 11.3% (95% CI, 9.5–13.3) (16). This well-characterized and manageable irAE profile, which appears favorable compared to conventional dual checkpoint inhibition, is a critical consideration for clinical decision-making. In the cadonilimab-lenvatinib combination study, the toxicity profile showed a lower incidence of severe irAEs typically associated with CTLA-4 inhibition and was instead dominated by adverse events commonly linked to lenvatinib (e.g., hypertension) or by complications related to the tumor burden itself (e.g., intestinal obstruction) (17).

#### 4.2.2 Mechanisms and management principles

The pathophysiology of irAEs stems from the non-specific activation of autoreactive T-cells and subsequent inflammatory damage to normal tissues. CTLA-4 inhibition primarily affects early T-cell activation in lymphoid organs, leading to a broader repertoire of activated T-cells, which explains the higher incidence of multi-organ irAEs with dual checkpoint blockade compared to PD-1 monotherapy. PD-1 blockade, operating mainly in peripheral tissues, is associated with more frequent tissue-specific toxicities.

Management of irAEs follows established guidelines (31) and is based on prompt recognition, grading, and intervention with corticosteroids (grade 2-4), or other immunosuppressants for steroid-refractory cases. For high-grade colitis, early initiation of infliximab is recommended. Endocrinopathies typically require hormone replacement therapy. Proactive patient education, regular monitoring of laboratory parameters (including thyroid function, liver enzymes, and cortisol), and multidisciplinary collaboration are essential for the safe administration of dual checkpoint blockade.

Given these complexities, the gynecologic oncologist must facilitate a shared decision-making process. This process should incorporate critical considerations such as tumor burden and the rate of disease progression, patient performance status and comorbidities, and individual preferences regarding risks and benefits.

### 4.3 Ensuring accurate biomarker testing

The effective implementation of precision oncology in endometrial cancer hinges on diagnostic accuracy. A critical challenge is the documented 3–7% discordance rate between mismatch repair (MMR) immunohistochemistry (IHC) and microsatellite instability (MSI) testing methods. This discordance can lead to both under-treatment and over-treatment (24). To mitigate this risk, expert consensus recommends the adoption of reflex testing protocols, such as confirmatory next-generation sequencing, following an abnormal IHC result, to enable more reliable treatment personalization (32).

### 4.4 Synthesis of efficacy and safety: informing clinical trade-offs

The integrated evidence from **Tables 1**, **2** establishes a clear efficacy-toxicity continuum for immunotherapy in dMMR/MSI-H EC. At one end, the conventional ipilimumab-nivolumab combination [as reported by Carlino et al. (12)] delivers the highest objective response rates (ORR 58%, **Table 2**) but imposes the greatest toxicity burden. This is characterized by nearly double the rate of grade ≥3 irAEs compared to monotherapy (23% versus 12%; see **Table 1**, Part B) and a notably higher incidence of high-grade gastrointestinal and hepatic events (**Table 1**, Part A). At the other end, pembrolizumab monotherapy [KEYNOTE-158 (4)] offers a more manageable safety profile, with fewer severe CTLA-4–associated toxicities, while maintaining clinically meaningful efficacy (ORR 48.5%, **Table 2**). Emerging agents such as the bispecific antibody cadonilimab, particularly in combination with lenvatinib, represent a potential intermediate option. Preliminary studies report encouraging activity (ORR 37.5–42.9%, **Table 2**) and indicate a distinct toxicity pattern that differs from existing therapies (16, 17). However, these findings require validation in randomized trials.

**Table 2:** Selected clinical trials of PD-1 and CTLA-4 targeting in advanced or recurrent endometrial carcinoma.

Trial/Agent	Phase	Design	Key inclusion criteria	Population	Key efficacy results (ORR)	Median PFS	Key safety results (Gr ≥3)	Reference/Status
dMMR/MSI-H Population								
Nivolumab + Ipilimumab	II	Basket, single-arm	Advanced dMMR/MSI-H non-colorectal cancers	EC subgroup: 26	63% (95% CI, 49-76)	NR	23% (irAEs)	Carlino et al. *JAMA Oncol.* 2025 (12)
Pembrolizumab(KEYNOTE-158)	II	Cohort, single-arm	Advanced MSI-H/dMMR tumors, ≥1 prior therapy	EC cohort: 79	48% (95% CI, 37-60)	13.1 months	12% (irAEs)	O’Malley et al. *J Clin Oncol.* 2022 (4)
Pembrolizumab + Chemotherapy (NRG-GY018)	III	Randomized, double-blind	First-line advanced dMMR EC	dMMR: 60	82.1% (95%CI, 72.9%-89.2%)	NR (HR 0.30)	9.7%(irAEs)	Eskander et al.*Nat Med*.2025 *(*21)
pMMR/MSS & Other population								
Pembrolizumab + Chemotherapy (NRG-GY018)	III	Randomized, double-blind	First-line advanced pMMR EC	pMMR: 291	72.3% (95%CI, 66.0%–78.1%)	11.1 months (HR 0.64)	9.7%(irAEs)	Eskander et al.*Nat Med*.2025 *(*21)
Lenvatinib+Pembrolizumab (KEYNOTE-775)	III	Randomized, open-label	Advanced pMMR EC, post-chemotherapy	pMMR: 411	32.4% vs 15.1%*	7.2 vs 3.8 months (HR 0.56)	TRAEs:90.1% vs 73.7%†	Makker et al. *J Clin Oncol*. 2023 (17)
Clear cell carcinoma								
Nivolumab + Ipilimumab	II	Nonrandomized, single-arm	Advanced clear cell carcinoma (endometrial & ovarian)	Total: 28 (EC: 6)	54% (95% CI, 35–71)	NR	35%(irAEs)	Gao et al. *JAMA Oncol*. 2025 (25)
Bispecific & Engineered antibodies								
Cadonilimab + Lenvatinib	II	Multicenter, single-arm	Advanced EC, progressed after platinum-based chemotherapy	32	37.5% (FAS)	NR (median FU 7.6 months)	TRAEs: 21.9%‡	Lan et al. SGO 2025 (17)
Ongoing trials								
NRG-GY025	III	Randomized, open-label	Recurrent dMMR/MSI-H EC	N/A	Primary Endpoints: PFS & OS (*Data Pending*)	N/A	N/A	NCT05112601 (Ongoing)
QLPPEC/GOG-003 (QL1706)	II	single-arm, open-label, multicenter	Advanced EC, post–PD-1/L1 therapy	N/A	ORR, PFS (*Data Pending*)	N/A	N/A	NCT06751277 (Ongoing)
QL1706 + Chemotherapy	II	single-arm, multicenter	1L Recurrent/Metastatic EC	N/A	PFS, ORR (*Data Pending*)	N/A	N/A	NCT06917092 (Ongoing)

CI, confidence interval; dMMR, mismatch repair-deficient; EC, endometrial carcinoma; FAS, full analysis set; FU, follow-up; HR, hazard ratio; irAEs, immune-related adverse events; MSI-H, microsatellite instability-high; NR, not reported; N/A, not applicable; ORR, objective response rate; OS, overall survival; PFS, progression-free survival; pMMR, mismatch repair-proficient; TRAEs, treatment-related adverse events.

*Compared to chemotherapy in KEYNOTE-775 trial.

†Any grade treatment-related adverse events.

‡Grade 3 or higher treatment-related adverse events.

This spectrum underscores that contemporary treatment selection is an exercise in calibrated trade-offs. The clinician must weigh the magnitude of anticipated benefit—considering factors such as tumor burden and disease kinetics, along with efficacy benchmarks from **Table 2**—against the acceptable threshold for toxicity, which is informed by patient performance status, comorbidities, and preference, as well as risk profiles detailed in **Table 1**. In the absence of validated predictive biomarkers, this careful balancing, grounded in the comparative data presented here, serves as the cornerstone of personalized therapeutic decision-making in advanced dMMR/MSI-H endometrial cancer.

## 5 Conclusions and future perspectives

The therapeutic landscape of dMMR/MSI-H endometrial carcinoma has been fundamentally reshaped by dual PD-1/CTLA-4 targeting, which now provides a multi-tiered strategy encompassing PD-1 monotherapy, conventional antibody combinations, and novel bispecific agents. Notwithstanding these advances, the integration of these regimens into a standardized treatment algorithm is hampered by the absence of head-to-head comparative trials and a dearth of predictive biomarkers beyond dMMR status.

Future progress hinges on addressing three pivotal challenges. First, the purported efficacy and safety advantages of novel constructs, particularly bispecific antibodies, must be rigorously evaluated in randomized controlled trials against the current standard of care. Second, a concerted translational effort is required to discover and validate biomarkers that can reliably stratify patients for treatment intensity. Third, the strategic expansion of combination immunotherapy into new clinical domains, notably in the first-line setting with chemotherapy, warrants careful exploration.

In this evolving and complex paradigm, the imperative for the clinical practitioner is to engage in a sophisticated decision-making process. This necessitates a critical appraisal of the available evidence, integration of comprehensive molecular profiling, and careful consideration of individual patient preferences and comorbidities to navigate the trade-offs between efficacy and toxicity, thereby personalizing therapy to optimize long-term outcomes.
